# [Retracted] LOXL1‑AS1 promotes thymoma and thymic carcinoma progression by regulating miR‑525‑5p‑*HSPA9*

**DOI:** 10.3892/or.2024.8789

**Published:** 2024-08-05

**Authors:** Jin Wang, Haihua Huang, Xiaomiao Zhang, Haitao Ma

Oncol Rep 45: 117, 2021; DOI: 10.3892/or.2021.8068

Following the publication of the above article, a concerned reader drew to the Editor's attention that certain of the cell invasion assay data featured in Figs. 2G and H, 5M and N, and 9K and L, and the tumor images shown in Fig. 6B, were strikingly similar to data appearing in different form in other articles written by different authors at different research institutes that had either already been published elsewhere prior to the submission of this paper to *Oncology Reports*, or were under consideration for publication at around the same time (some of which have been retracted).

In view of the fact that certain of these data had already apparently been published prior to the submission of this article for publication, the Editor of *Oncology Reports* has decided that this paper should be retracted from the Journal. The authors were asked for an explanation to account for these concerns, but the Editorial Office did not receive a satisfactory reply. The Editor apologizes to the readership for any inconvenience caused.

